# One-Step and Real-Time Detection of microRNA-21 in Human Samples for Lung Cancer Biosensing Diagnosis

**DOI:** 10.1021/acs.analchem.2c02895

**Published:** 2022-10-11

**Authors:** Olalla Calvo-Lozano, Pablo García-Aparicio, Lajos-Zsolt Raduly, Maria Carmen Estévez, Ioana Berindan-Neagoe, Manuela Ferracin, Laura M. Lechuga

**Affiliations:** † Nanobiosensors and Bioanalytical Applications Group (NanoB2A), Catalan Institute of Nanoscience and Nanotechnology (ICN2), CSIC, CIBER-BBN and BIST, Campus UAB, 08193 Bellaterra, Barcelona, Spain; ‡ Research Center for Functional Genomics, Biomedicine and Translational Medicine, University of Medicine and Pharmacy “Iuliu Hatieganu”, Gheorghe Marinescu 23, 400337 Cluj-Napoca, Romania; § Department of Experimental, Diagnostic and Specialty Medicine (DIMES), University of Bologna, Via S. Giacomo 14, 40126 Bologna, Italy

## Abstract

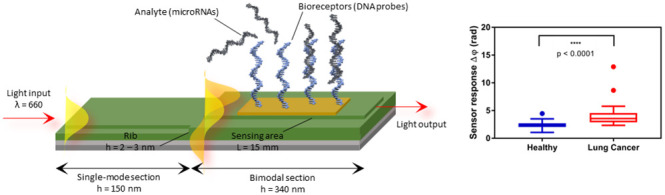

The rapid diagnosis of cancer, especially in its early stages, is crucial for on-time medical treatment and for increasing the patient survival rate. Lung cancer shows the highest mortality rate and the lowest 5-year survival rate due to the late diagnosis in advanced cancer stages. Providing rapid and reliable diagnostic tools is a top priority to address the problem of a delayed cancer diagnosis. We introduce a nanophotonic biosensor for the direct and real-time detection in human plasma of the microRNA-21-5p biomarker related to lung cancer. The biosensor employs a silicon photonic bimodal interferometric waveguide that provides a highly sensitive detection in a label-free format. We demonstrate a very competitive detectability for direct microRNA-21-5p biomarker assays in human plasma samples (estimated LOD: 25 pM). The diagnostic capability of our biosensor was validated by analyzing 40 clinical samples from healthy individuals and lung cancer patients, previously analyzed by reverse-transcription quantitative polymerase chain reaction (qRT-PCR). We could successfully identify and quantify the levels of microRNA in a one-step assay, without the need for DNA extraction or amplification steps. The study confirmed the significance of implementing this biosensor technique compared to the benchmarking molecular analysis and showed excellent agreement with previous results employing the traditional qRT-PCR. This work opens new possibilities for the true implementation of point-of-care biosensors that enable fast, simple, and efficient early diagnosis of cancer diseases.

Lung cancer (LC) presents the highest incidence and mortality rates worldwide. In 2020, 2.206.771 new cases and 1.796.144 deaths were reported, representing close to 1 in 5 (18.4%) cancer-related deaths.^1^ In the last few years, LC has caused more deaths than breast, prostate, colorectal, and brain cancers combined, being the top cancer death in men and the second one in females, after breast cancer. Besides, LC has a 5-year survival rate of 19%, second only to pancreatic cancer, which is the cancer with the poorest prognosis.^2^ Most LCs are asymptomatic (or present common symptoms such as cough, anorexia, fatigue, or dyspnea).^3^ Thus, individuals are not diagnosed promptly, leading to long-term sequels like advanced cancer stages characterized by metastasis in which treatment is not able to effectively injure the tumor.^4^

Nowadays, the techniques for LC diagnosis rely on imaging methodologies like chest radiography, contrast-enhanced computed tomography (CT), and positron emission tomography. These techniques show low sensitivity, are costly, and imply radiation generation.^5^ In addition, they are only useful when the tumor is visible enough. For optimizing the diagnosis and treatment, they should be combined with other molecular techniques such as cytology samples and small biopsies.^3^ Clinical diagnosis based on detecting biomarkers such as proteins or genetic material in body fluids such as urine, saliva, or blood is boosting the cancer diagnosis. The biomolecular analysis enables the identification of a cancerous development even in the early stages, before tumor presence, by employing a noninvasive and inexpensive method^5^ permitting rapid medical treatment and increasing the cancer survival rate. To diagnose LC, a large list of different biomarkers has been described such as proteins (i.e., NSE, CEA, and CYFR A-21),^6^ epigenetic events like DNA methylation (CDO1 gene, ZNF177 gene),^7^ DNA mutations (K-RAS gene, PTEN gene),^8^ or circulating microRNAs (microRNA-21-5p, microRNA-205-5p, and microRNA-210-3p) among others.^9^ Specifically, microRNAs, which are short and single-stranded noncoding RNAs (∼22 nucleotides), play an important role in the modulation of several biological processes, such as cell cycle control, apoptosis, and differentiation, being involved in the tumorigenesis process.^9^ The expression of a specific microRNA signature can determine the type of cancer according to the tissue^10^ and its development and progression stage (early or late stage, metastasis...).^11^ At present, microRNA detection is based on reverse-transcription quantitative polymerase chain reaction (qRT-PCR) or digital PCR techniques, Northern Blot, and high-throughput sequencing (i.e., microarrays or next generation sequencing). Despite the reliability and feasibility of these established techniques, they might require large amounts of purified sample, labels, and long incubation times that imply a laborious preparation and rigorous experimental conditions. Moreover, the lack of standardized protocols for microRNA extraction from blood can introduce a high variability, leading to noncomparable analyses.^12^ Therefore, efficient and reliable detection strategies for circulating microRNA quantification are crucial. Rapid and simple detection of microRNA biomarkers in the blood of cancer patients can not only facilitate prompt treatment but also potentially increase the survival rate and decrease the mortality rate.

Biosensors constitute an excellent opportunity to develop integrated devices that enable fast and accurate clinical diagnosis. In particular, optical evanescent wave-based technology offers label-free and real-time quantitative analysis with high sensitivity and a remarkable potential for miniaturization in point-of-care devices.^13^ Evanescent wave biosensors are excellent tools for the detection of microRNAs due to the minimal sample preparation, absence of amplification steps, high sensitivity, fast outcome, and multiplexing capability. Several optical biosensors have been proposed to detect circulating microRNAs for clinical diagnosis. The most employed biosensors are based on the surface plasmon resonance, reaching limits of detection (LOD) in the pM–fM range when they include amplification steps with antibodies,^14^ gold nanoparticles,^15^ or catalytic reactions.^16^ Among silicon photonic biosensors, microring resonators^17^ and a Mach Zehnder interferometer^18^ have been also employed for microRNA detection, reaching LOD in the nM range.

We present an advanced nanophotonic biosensor based on bimodal waveguide interferometers (BiMW).^19^ The working principle of a BiMW biosensor relies on the evanescent wave, an electromagnetic field generated when polarized light propagates through a waveguide by total internal reflection. The evanescent wave arises when part of the energy is not totally confined and penetrates in the external medium up to hundreds of nanometers. The evanescent wave is highly sensitive to changes in the refractive index of the medium and in close proximity to or on the waveguide’s surface. In the case of the BiMW biosensor, a polarized monochromatic light is coupled into the bimodal waveguides and propagated along its core, allowing the excitation of two light modes. These modes produce an interference pattern that is dependent on the local refractive index at the waveguide surface. Any event at the sensor surface, such as the binding of an analyte to its specific receptor, results in a change in the local refractive index, which produces a phase shift between the two modes, and hence, an interference pattern that can be monitored instantaneously. The BiMW design is based on a linear straight waveguide with a step junction, its fabrication being simpler than the one employed for other interferometers like Mach Zehnder or Young designs, characterized by Y-junctions and two independent arms.^20^ In addition, BiMW sensor chip fabrication is performed by standard microelectronic technology that enables reducing fabrication costs and increasing reproducibility and reliability. The BiMW biosensor has previously demonstrated its potential for clinical diagnostics in several areas, including infectious diseases,^21,22^ cancer diagnosis,^23^ and endocrinology,^24^ enabling direct, sensitive, and reliable detection of bacteria, microRNAs, and hormones, respectively. So far, we had not yet evaluated the effect of complex fluids like serum and plasma with this device. Herein, we have implemented and validated this powerful biosensor for the detection of circulating microRNAs released in the blood of non-small cell lung cancer (NSCLC) patients. By functionalizing the sensor surface with specific DNA probes, we carried out a complementary hybridization assay to identify the presence of a relevant microRNA biomarker (microRNA-21-5p) present in plasma. The BiMW sensor enables not only direct and real-time quantitative analysis but also provides a rapid detection and diagnosis (less than 45 min) without the need for amplification steps or sample pretreatment, contrary to the conventional qRT-PCR. This methodology represents a significant step forward for the use of optical biosensor devices for complex fluid analysis and particularly for an efficient cancer diagnosis.

## Experimental Section

### Chemical and Biological Reagents

All chemical reagents are described in the Supporting Information (SI). The DNA probe and synthetic microRNAs employed for the optimization of the hybridization complementary assay were purchased from IBIAN Technologies (Zaragoza, Spain) and are summarized in Table 1. Human pooled plasma was purchased from Innovative Research (Michigan, US).

**Table 1:** Nucleotide Sequences Employed in This Work

nucleotide sequences	sequence (5′ → 3′)
DNA probe miRNA-21	[Thiol]TTT TTT TTT TTT TTT TCA ACA TCA GTC TGA
microRNA-21-5p (target)	UAGCUUAUCAGACUGAUGUUGA
microRNA-210-3p (control)	CUGUGCGUGUGACAGCGGCUGA

### BiMW Biosensor Device

The biosensor employed is an in-house designed and assembled BiMW device that incorporates all the optical and microfluidic components. A detailed description is provided in the SI and Figure S1.

### Biofunctionalization of the Sensor Chips with the DNA Capture Probe

The sensor chips were cleaned and silanized with APTES-PDITC (as described in the SI) and placed on the experimental set-up for the in situ immobilization of the thiolated DNA probes to the R-NCS groups (Figure S2A). DEPC-H2O water was kept as running buffer, and the immobilization solution was flown at a constant rate of 3 μL min^–1^. The immobilization solution contained a mixture of a DNA SH-T15-miRNA21 probe and SH-PEG-COOH spacer (total thiol 2 μM, molar ratio of DNA/spacer of 1:1) in phosphate immobilization buffer. Before injection, the immobilization solution was incubated with 0.1 μM TCEP solution in constant agitation for 20 min at 36 °C.

To avoid nonspecific adsorptions from plasma samples, a blocking step was included by employing BSA 20 mg mL^–1^ diluted in PBS which was injected over the sensor chip after the immobilization step at 5 μL min^–1^. Finally, the sensor chips were kept under a continuous flow of SSC-P (SSC 2.5X + 0.5% Tween 20 + 10 mM CHAPS) at 10 μL min^–1^. Figure S2B shows a sensorgram of the two-step reaction involved in the covalent coupling of both, DNA probes and BSA, to the Si_3_N_4_ sensor surface.

### Complementary Hybridization Assay Performance

**Figure 1:**
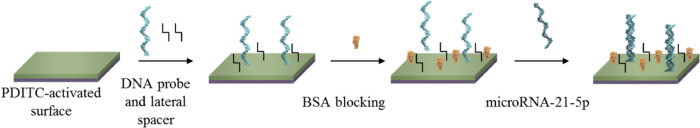
BiMW sensor biofunctionalization for microRNA-21-5p detection. Representation of the three main steps in the development of a complementary hybridization assay for the detection of microRNA-21-5p.

Calibration curves in standard buffer conditions were obtained by flowing different microRNA-21-5p solutions (from 0.5 to 100 nM, 150 μL) dissolved in SSC 5X buffer (0.75 M in NaCl, 0.075 M in sodium citrate) over the BiMW biofunctionalized biosensor surface at a 10 μL min^–1^ rate, using SSC 5X as running buffer. Calibration curves were obtained by analyzing different microRNA-21-5p concentrations in triplicate. Calibration curves in plasma were generated by flowing different concentrations of microRNA-21-5p (ranging from 0.5 to 100 nM) spiked in undiluted human plasma over the BiMW sensor surface at a flow rate of 10 μL min^–1^, using SSC-P (SSC 2.5X + 0.5% Tween 20 + 10 mM CHAPS) as running buffer. In all cases, DNA probe/microRNA interaction was disrupted by injecting a 5 mM NaOH regeneration solution for 30 s at a constant flow rate, allowing the reuse of the sensor chips for 10 cycles without affecting assay performance. Figure 1 shows a schematic representation of the complete biofunctionalization protocol.

### Clinical Sample Collection

A total of 20 patients from the “Leon Daniello” Pneumophtisiology Clinical Hospital Cluj-Napoca, Romania diagnosed with NSCLC were enrolled in our study. Besides the LC patients, we enrolled 20 healthy subjects in our study for blood sample donation (controls) (Table 2). The plasma samples were isolated by centrifugation at 4200 rpm/10 min at room temperature. From each patient blood, samples were collected according to the hospital protocol and the informed consent approved by the Ethical Committee no. 438/2016.

**Table 2:** Lung Cancer Patients and Healthy Subjects Included in the Study

sample type	no. samples	cancer stage	plasma origin
plasma	20 (50%)	IIIA-(25%)/IIIB-(50%)/IV-(25%)	LC patients (NSCLC)
plasma	20 (50%)		healthy subjects

### RNA Extraction and qRT-PCR

Total RNA was extracted and isolated from plasma samples using a Plasma/Serum Circulating and Exosomal RNA Purification Kit (Norgen Biotek Corp., Ontario, Canada) according to the manufacturer’s protocol. The RNA concentration was measured using a NanoDrop-1000 spectrophotometer (ThermoFisher Scientific, Massachusetts, US). Total RNA concentration was ranged to 25 ng μL^–1^. microRNA-21-5p was selected for the plasma microRNA profile analysis by qRT-PCR. The cDNA synthesis was performed using 7.5 μL of reverse transcription mixture containing 0.72 μL of RT primer, 25 ng of total RNA and 0.5 μL of MultiScribe reverse transcriptase, 0.75 μL of reverse transcription buffer (10×), 0.075 μL of dNTPs (100 mM), and 0.1 μL of RNase inhibitor according to a TaqMan MicroRNA reverse transcription kit (Applied Biosystems, Massachusetts, US) protocol. The cDNA mixture was incubated in PCR tubes for 16 °C 30 min, 42 °C 30 min, and 85 °C 5 min. qRT-PCR was performed in a total volume of 10 μL using 5 μL of 5.5 TaqMan Fast Advanced Master Mix and 0.47 μL of primer for each microRNA in a ViiA7 (Applied Biosystems, Massachusetts, US) PCR machine. The reactions were set up as follows: the initial step includes the UNG incubation at 50 °C for 2 min and polymerase activation at 95 °C for 20 s, followed by 40 cycles of 95 °C for 1 s (denature) and 60 °C (anneal/extend) for 20 s. The relative expression level was calculated using 2^–ΔΔCT^ (fold change).^25^ A median Ct across all samples was chosen as a calibrator and miR-16-5p as the endogenous control. Thermo Fisher microRNA Primer Assay ID: hsa-miR-21-5p #000397; hsa-miR-16-5p #000391.

### Data Analysis

The real-time sensorgrams were processed extracting the final response (Δφ) after signal stabilization once the whole sample volume has passed through the flow cell. Details of the fitting curves are described in the SI. Statistical analysis assessing the differences between healthy and LC groups was analyzed with the Mann–Whitney test considering a *p*-value < 0.05 to be statistically significant. The correlation between the BiMW biosensor and qRT-PCR was analyzed by the Spearman test considering a *p*-value < 0.05. In order to evaluate the diagnosis capabilities of both the BiMW biosensor and qRT-PCR, receiver operating characteristic (ROC) curves were also performed. Statistical analysis to discover any significant relationship between sensor response and cancer stages was done with the Kruskal–Wallis test, considering a *p*-value < 0.05.

## Results and Discussion

### Biosensor Assay and Analytical Characterization

**Figure 2:**
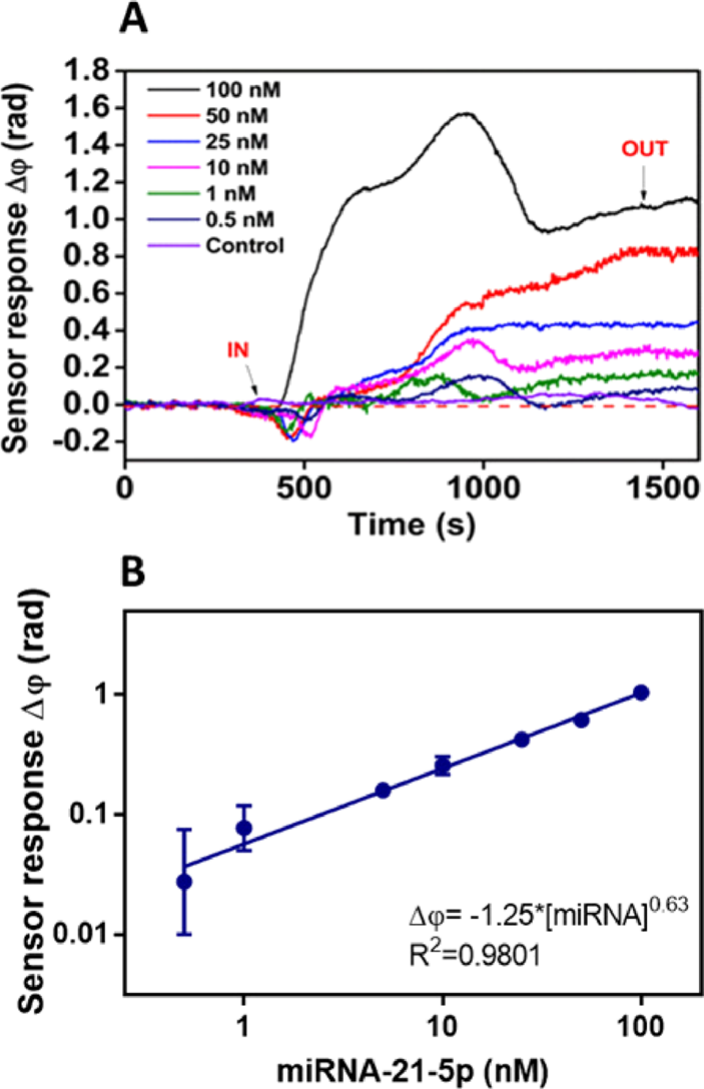
BiMW-based microRNA-21-5p hybridization assay in buffer. (A) Real-time sensorgrams showing the specific interaction of the DNA probe with different microRNA-21-5p concentrations. Nonspecific microRNA-210-3p (control) was measured at a concentration of 100 nM. (B) Calibration curve in hybridization buffer (SSC 5X) (log–log display). Each signal corresponds to the mean ± SD of triplicate measurements. IN (*t* ∼ 500 s) and OUT (*t* ∼ 1500 s) arrows indicate the start and end time of the injection, respectively.

In order to demonstrate and evaluate the performance of the BiMW biosensor, an assay strategy based on direct hybridization was designed, employing a synthetic DNA probe fully complementary to the microRNA-21-5p as the capture probe, thus providing the required specificity. Figure 2 shows the biosensor signals obtained for microRNA-21-5p detection. Slight signal fluctuations were observed, while the sample was entering the cell, attributed to the running buffer and the modulation method employed to convert the interferometric signal into a linear one (see Section 2.1 in the SI). However, this was corrected once all the sample had flown, resulting in stabilized signals. The sensor response (Δφ, rad) gradually increased as the microRNA concentrations were higher. The representation of the log–log variables adequately fitted to a linear curve (see Figure 2B and Section 3 in the SI) for the range of microRNA concentrations analyzed (i.e., 0.5–100 nM), being possible to establish an estimated LOD = 34 pM (*R*^2^ = 0.9801). Even though there is not a consensus about the concentration of microRNA in plasma due to the lack of standardization in RNA extraction and the variation between quantification methodologies, some studies suggest that the microRNA concentration in human plasma might lie in the range of 10^5^–10^8^ copies mL^–1^ (i.e., fM–nM).^26^ According to these LOD, the performance of our biosensor may provide enough analytical sensitivity for LC diagnosis.

The assay specificity was also evaluated to guarantee the absence of nonspecific interactions of other microRNAs involved in LC (i.e., microRNA-210-3p). As we can observe in Figure 2A, microRNA-210-3p (control) interacted neither with the sensor surface nor with the DNA probe (i.e., sensor response Δφ = 0 rad after signal stabilization). Net sensor response confirms the absence of cross-reactivity and ensures that the signals come exclusively from specific microRNA–complementary DNA probe interactions.

The reproducibility of the assay was evaluated through the interassay variability (replicates in different sensor chips). The CV% values obtained for buffer conditions were below the maximum variability recommended for clinical analysis (15–20%)^27^ (slightly higher for the LOD) (Table 3), verifying the good reproducibility and suitability of this hybridization assay.

**Table 3:** Interassay Variability for Buffer and Undiluted Plasma Calibration Curves (CC)^a^

matrix	parameters		
buffer	*A*	*b*	LOD, pM
CC1	–1.27	0.64	43
CC2	–1.18	0.59	20
mean ± SD	–1.23 ± 0.06	0.61 ± 0.03	31.5 ± 16.2
%CV	5.3%	5.7%	51%

plasma	*A*	*b*	LOD, pM
CC1	–0.96	0.56	18
CC2	–1.00	0.58	23
mean ± SD	–0.98 ± 0.03	0.57 ± 0.01	20.5 ± 3.5
CV%	3.4%	2.6%	17%

a Eq: log((Δφ) = *A* + *b* · log ([miRNA]) → Δφ = *A*·[miRNA]^*b*^.

### Human Plasma Effect on the Assay Performance

To apply the described biosensor methodology for the analysis of LC patients’ plasma samples, it is crucial to consider the influence of the plasma matrix on the sensor surface and the hybridization event. Plasma contains high amounts of proteins and other compounds that could generate nonspecific interactions or hinder the DNA probe–microRNA interaction (i.e., untreated biofunctionalized sensor chip resulted in extremely high signals of around Δφ ≈ 40 rad). Therefore, we combined the use of blocking agents (added to the surface to increase its biocompatibility and hydrophilicity) with mixtures of additives that help shield nonspecific interactions (see Figure S3). According to the conditions tested, we decided to employ a combination of BSA 20 mg mL^–1^ as the blocking agent and Tween 20 0.5% + CHAPS 10 mM, nonionic, and zwitterionic surfactants, respectively, as the best suitable additives that overall successfully removed all nonspecific interactions from human plasma (Δφ ≈ 0 rad).

**Figure 3:**
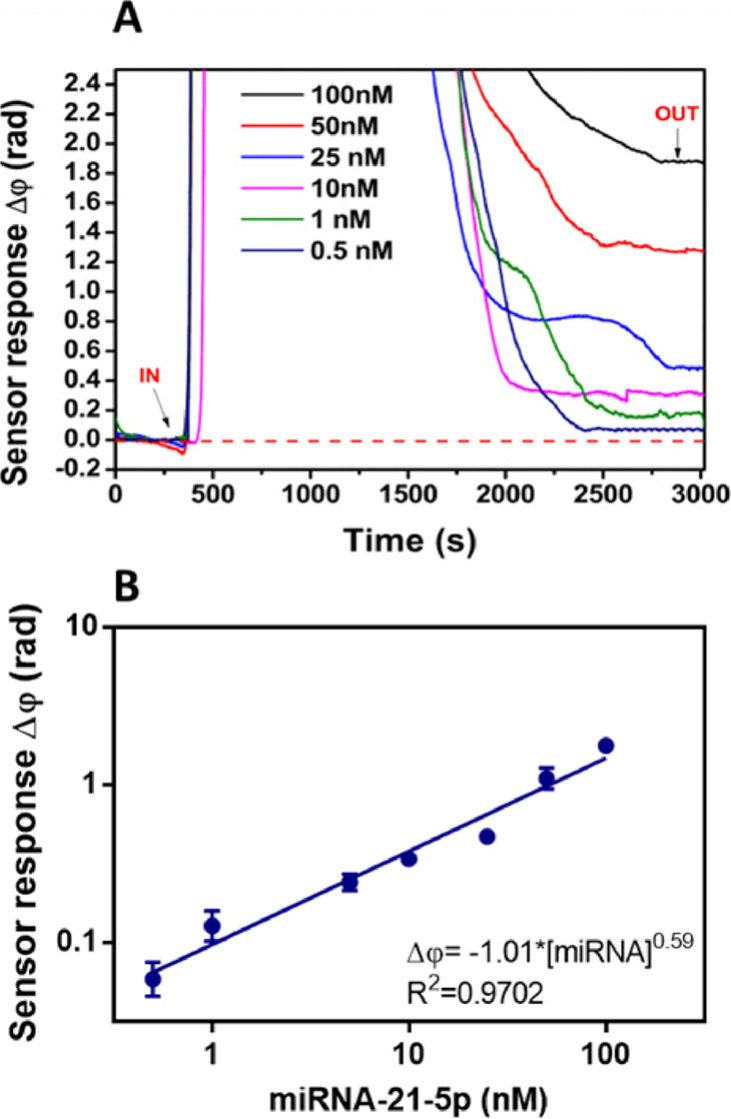
BiMW-based microRNA-21-5p hybridization assay in plasma. (A) Real-time sensorgrams showing the specific interaction of the DNA probe with different microRNA-21-5p concentrations spiked in human plasma. (B) Calibration curve in human plasma (log–log display). Each signal corresponds to the mean ± SD of triplicate measurements. IN (*t* ∼ 500 s) and OUT (*t* ∼ 3000 s) arrows indicate the start and end time of the injection, respectively.

Pooled human plasma was spiked with different microRNA-21-5p concentrations in the range from 0.5 to 100 nM (Figures 3 and S4 in the SI). The calibration curve performed in plasma showed an estimated LOD of 25 pM (*R*^2^ = 0.9701). The similar LOD value obtained compared with the one in standard buffer conditions (34 pM) reveals that the biological fluid did not compromise the assay sensitivity, not affecting the sensor surface or the DNA probe and its capabilities to hybridize. The assay in plasma showed a similar reproducibility to the one observed in standard buffer conditions (see Table 3) which confirms the suitability of the designed strategy for the accurate detection of microRNA-21-5p with the BiMW biosensor-based assay. Under these optimized conditions, the biosensor assay was further evaluated with real clinical samples.

### Clinical Validation of BiMW-Based microRNA-21-5p Assay

**Figure 4:**
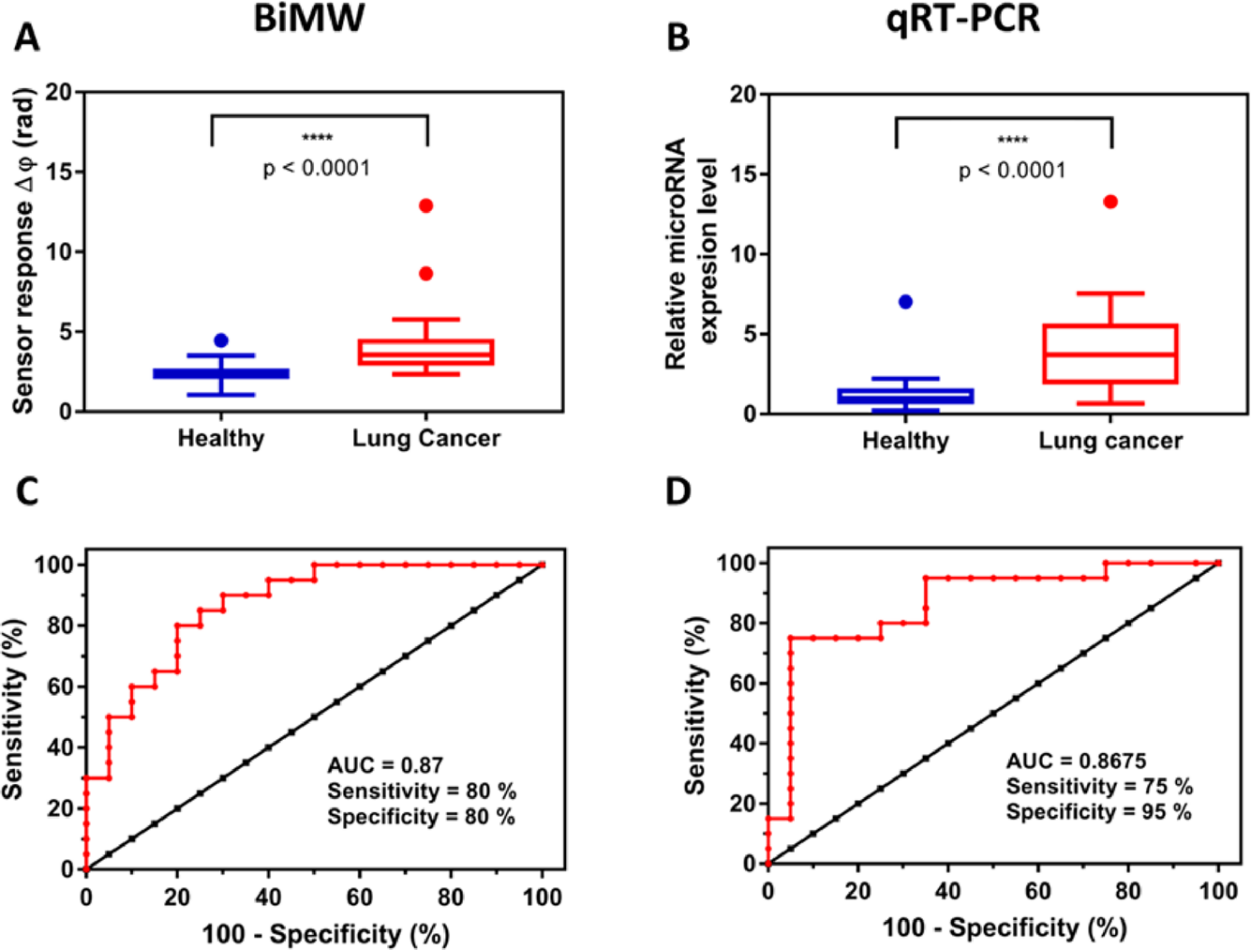
microRNA-21-5p detection in clinical samples based on (A) BiMW sensor and (B) qRT-PCR in 20 healthy and 20 LC individuals. Mann–Whitney test *p*-value < 0.0001. Outliers are also shown. ROC curve analysis of (C) BiMW technology and (D) qRT-PCR. AUC, sensitivity, and specificity values are reported.

We have assessed a set of 40 plasma clinical samples from the Research Center for Functional Genomics, Biomedicine and Translational Medicine (Romania). The collection consisted of 20 LC plasma samples and 20 negative samples from healthy donors. LC plasma samples were collected from patients previously diagnosed with LC, specifically, NSCLC through bronchoscopy. All samples were previously validated by qRT-PCR in the Research Center for Functional Genomics, Biomedicine and Translational Medicine, with a positive result for microRNA-21-5p (Table S1). All the samples were analyzed with the BiMW biosensor, and a statistical comparison between healthy and LC individuals was carried out. Figure 4A presents the distribution of the biosensor response obtained by using our hybridization assay, showing statistical significance differentiation between healthy [median = 2.368] and LC patient [median = 3.563] (*p*-value < 0.0001; *p*-value < 0.05). Additionally, an ROC curve was performed to evaluate the diagnostic specificity and sensitivity of the BiMW biosensor technology (Figure 4C). The area under curve (AUC) value determines the capability of a diagnostic test to discriminate between healthy and patients by considering 1 as an excellent and 0.5 as random diagnosis. The BiMW AUC outcome was 0.87 (CI95%, 0.7616–0.9784), reflecting an appropriate diagnosis capability with a sensitivity of 80% and specificity of 80%.

The BiMW biosensor performance was qualitatively compared to the standard technique qRT-PCR. Results are shown in Figure 4. As can be observed, qRT-PCR-based microRNA quantification was able to discriminate between healthy [median = 0.99] and LC patients [median = 3.7] in a statistically significant manner (*p*-value < 0.0001) (Figure 4B). ROC curves determined that, as the BiMW biosensor, the qRT-PCR methodology presented acceptable LC detection capabilities (AUC 0.8675 (CI95%, 0.75–0.985), sensitivity 75%, and specificity 95%) (Figure 4D). An additional correlation analysis was carried out by the Spearman test, showing a significant relationship between the quantification value obtained with qRT-PCR and the signal obtained with the BiMW biosensor for most of the samples, which might reflect the reliability of the BiMW biosensor assay (Spearman coefficient = 0.373, *p*-value = 0.018, and *p*-value < 0.05) (data not shown). These results corroborate the competitive performance of our BiMW technology compared with the benchmarked qRT-PCR. Moreover, the BiMW biosensor can provide highly accurate detection of microRNA in less than 45 min, with diagnostic reliability equivalent to qRT-PCR, without previous amplification or purification steps, revealing the potential of the nanophotonic waveguide interferometer biosensor technology for LC clinical diagnosis.

**Figure 5:**
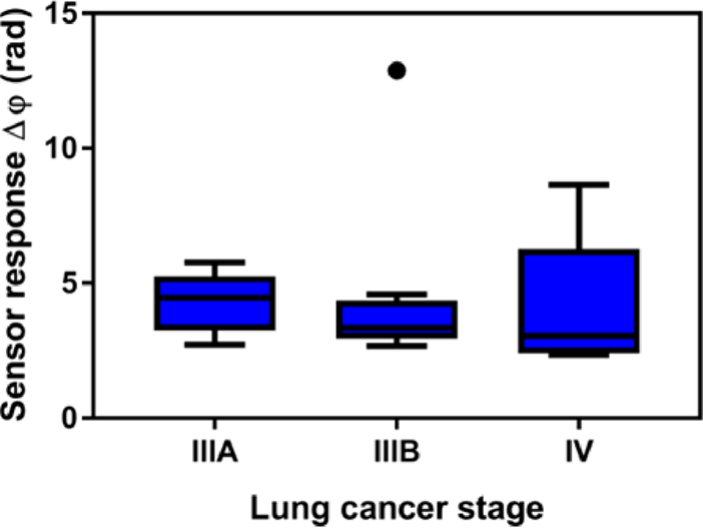
Correlation cancer stage vs microRNA-21-5p concentration. Sensor signal for the 20 LC plasma samples from individuals in different oncological stages (IIIA, IIIB, and IV). Kruskal–Wallis test (*p*-value = 0.4833; *p*-value > 0.05). Outliers are also shown.

Finally, to test the capabilities of the BiMW biosensor, a study with the 20 clinical samples from LC patients was carried out to assess a possible correlation between the cancer stage and the levels of microRNA-21-5p in the plasma. To stratify patients according to their oncological status, the TNM classification was used in which the size and extent of the primary tumor (T), the number of nearby lymph node invasion (N), and the absence or presence of metastasis (M) were analyzed (Table S1 in the Supporting Information). We evaluated 20 plasma samples from different LC stages [IIIA (*n* = 5), IIIB (*n* = 10), and IV (*n* = 5)] (Figure 5).

As Zhang et al. reported,^28^ the overexpression of microRNA-21-5p, and therefore its concentration in body fluids, increases depending on the TNM stage, being higher in advanced TNM cancers. Nevertheless, Figure 5 reveals that despite the capability of the BiMW to diagnose LC, no conclusive evidence could be extracted regarding a possible correlation between the LC stage and the epigenetic response reflected in the microRNA-21-5p concentration in plasma. Statistical analysis shows a nonsignificant difference in the microRNA concentration between average groups (IIIA [4.290]; IIIB [4.383]; IV [4.074], with a *p*-value = 0.4833). The limited number of samples and above all the difficulty in collecting early-stage LC samples, since all LC samples presented advanced TNM stage LC (III and IV), hinder the capability to prognosticate and prompt diagnose this type of cancer with our biosensor. We acknowledge the necessity of completing this study with an extended number of samples and a more variety of LC samples (i.e., from stage 0 to stage IV). Despite the mentioned limitations, the BiMW technology exemplifies the benefit that a quantitative microRNA biosensor assay can provide for monitoring cancer progression.

## Conclusions

We have demonstrated a novel BiMW biosensor methodology for the fast, direct, and quantitative identification of the LC -related microRNA-21-5p biomarker in human plasma. Our advanced biosensor, based on a nanowaveguide interferometric technology, offers high sensitivity and label-free analysis in real time. The complementary hybridization strategy consisting of covalent immobilization of DNA probes that provides the assay specificity reached excellent LOD in plasma (pM range) enabling one-step detection and quantification of microRNA-21-5p in LC samples. A clinical validation with healthy and LC patients (*n* = 40) showed excellent discrimination between healthy and cancer samples (*p*-value < 0.0001), with a performance similar to the one of established techniques such as qRT-PCR and with the additional advantage of avoiding RNA extraction or amplification steps. In addition, the clinical validation demonstrated diagnostic sensitivity and specificity of 80% in both cases. We have also conducted a small-scale study to assess the capabilities of our strategy for early diagnosis of LC, although clinical samples at stages 0, I, or II would be necessary to carry out a complete analysis and to extract relevant conclusions.

The presented work represents a relevant step toward the implementation of this biosensor for clinical diagnosis. The obtained results place our biosensor device as an accurate and reliable tool for rapid and direct detection of microRNA, becoming a potential alternative tool for early cancer diagnosis and treatment in a noninvasive manner and with great perspectives in clinical practice.

## Supporting Information Available

Description of the BiMW biosensor device, and details of reagents and buffers, biosensor chip biofunctionalization, data analysis, and additional results for the validation of the methodology (PDF)
