# Supplementary material for: One-Step and Real-Time Detection of microRNA-21 in Human Samples for Lung Cancer Biosensing Diagnosis
Source: Anal Chem. 2022 Oct 11;94(42):14659–65. doi: 10.1021/acs.analchem.2c02895 (PMC9607850; doi:10.1021/acs.analchem.2c02895)
Supplement: Supplementary file 1 — ac2c02895_si_001.pdf [file ac2c02895_si_001.pdf]

Supporting Information

One-step and real-time detection of microRNA-21 in human samples for lung cancer biosensing diagnosis

Olalla Calvo-Lozano[1], Pablo García-Aparicio[1], Lajos-Zsolt Raduly[2], Maria Carmen Estévez[1*], Ioana Berindan-Neagoe[2], Manuela Ferracin[3] and Laura M. Lechuga[1]

[1] Nanobiosensors and Bioanalytical Applications Group (NanoB2A), Catalan Institute of Nanoscience and Nanotechnology (ICN2), CSIC, CIBER-BBN and BIST, Campus UAB, 08193 Bellaterra, Barcelona, Spain

[2] Research Center for Functional Genomics, Biomedicine and Translational Medicine, University of Medicine and Pharmacy "Iuliu Hatieganu", Gheorghe Marinescu 23, 400337, Cluj-Napoca, Romania.

[3] Department of Experimental, Diagnostic and Specialty Medicine (DIMES), University of Bologna, Via S. Giacomo 14, 40126, Bologna, Italy

** Corresponding author:* *M.-Carmen Estevez: mcarmen.estevez@icn2.cat*

Table of contents

1. Chemical and biological reagents S-2
2. BiMW interferometer sensor
   2.1. The BiMW device S-2
   2.2. BiMW sensor chips cleaning and silanization S-3
3. Data analysis S-3
4. Figures S1-S4 S-4
5. Lung cancer clinical samples. Table S-1 S-6
6. References S-7

1. Chemical and biological reagents

Solvents used for sensor chips' cleaning - acetone 99.5%, ethanol 99% and methanol 99%, hydrochloric acid (HCl, 37 %), nitric acid (HNO_3, 65 %) -were purchased to Panreac Applichem (Barcelona, Spain). Main salts for buffer preparation and biofunctionalization, anhydrous toluene 99.8%, N, N-dimethylformamide anhydrous ≥99.8%, (DMF), anhydrous pyridine 99.8%, crosslinking molecule p-phenylene diisothiocyanate 98% (PDITC), (3-aminopropyl)triethoxysilane ≥98%, (APTES), N, N-diisopropylethylamine (DIPEA), diethyl pyrocarbonate (DEPC), bovine serum albumin (BSA), Tween 20, were acquired from Sigma-Aldrich (Darmstadt, Germany). Lateral spacer SH-PEG-COOH, (MW 2000 g mol^{-1}) was purchased from Laysan Bio (Alabama, US). 3-[3-Cholamidopropyl)dimethylammonio]-1-propane sulfonate (CHAPS) was purchased from Avanti Polar Lipids (Alabama, US). Bond-Breaker™ TCEP Solution (tris(2-carboxyethyl)phosphine hydrochloride solution) was purchased from ThermoFisher (Massachusetts, US). The buffers employed were the following: sodium citrate buffer (SSC) 20X (3 M NaCl, 0.3 M sodium citrate, EDTA 4 mM, pH 7.4), phosphate immobilization buffer 50 mM (NaCl 0.84 M, KCl 13.5 mM, Na_2HPO_4 50 mM, KH_2PO_4 9 mM, EDTA 10 mM, pH 7.1), phosphate buffer saline (PBS) 10 mM (NaCl 0.137 M, KCl 2.7 mM, Na_2HPO_4 10 mM, KH_2PO_4 1.8 mM, pH 7).

To work under RNase-free conditions, all the labware used was sterilized, and buffer solutions were prepared with milli-Q H_2O treated with 0.1% DEPC and autoclaved at 121ºC for 1 hour.

MicroRNA sequences for probe and target design were extracted from the miRBase database (http://www.mirbase.org). DNA probe sequences are complementary to microRNA-21-5p and incorporate a thiol (SH-) functional group at the 5'-end to enable coupling with PDITC-activated monolayer in silicon nitride (Si_3N_4) surfaces. In addition, a spacing region that consists of a 15 thymines (polyT$_{15}$) sequence is placed between the functional group and the matching region for enhancement of target accessibility.

2. BiMW interferometer sensor

2.1. BiMW device

The BiMW sensor chips (3 cm × 1 cm; **Figure S1A**) containing an array of 20 independent bimodal waveguides were fabricated at wafer-scale in Si_3N_4 technology in a Clean Room facility (at the Institute of Microelectronics IBM-CNM-CSIC, Barcelona, Spain), as previously described Zinoviev et al. [1]. The working principle of the BiMW sensor is based on the behavior of light propagating through a waveguide where only the fundamental and first modes of transverse electric polarized light can be

propagated. Briefly, light from a polarized diode laser (λ = 660 nm) (Hitachi, Tokyo, Japan) is confined in a rib waveguide designed to support a single (fundamental) mode through the waveguide core (150 nm thickness). After a certain distance, this fundamental mode is coupled into a bimodal section (300–350 nm thickness) through a step junction that permits the additional excitation of the first propagating mode. A sensing window is opened along the bimodal section of the waveguide where the bioreceptors are immobilized and detection takes place (**Figure S1B**). The two modes travel until the end of the waveguide, generating different evanescent field profiles that decay within the external medium. Any change in the refractive index near the sensor surface, such as the one induced by the binding (or detachment) of any molecule, differently affects the propagating modes and results in an interferometric phase shift ($\Delta\varphi$) between the two modes, modifying the intensity distribution at the sensor chip output. The intensity is recorded by a two-sectional photodetector (Hamamatsu Photonics, Hamamatsu, Japan) and processed through an acquisition card. An all-optical phase modulation method based on a trigonometric algorithm is applied [2], transforming the interference signal into a linear one, being able to quantify the phase shifts between both modes continuously. The incorporation of a Peltier thermoelectric cooler (Peltier element TEC3-2.5) (Thorlabs, New Jersey, US) and a temperature controller behind the sensor chip avoid temperature fluctuations that affect the intrinsic sensitivity of the BiMW biosensor, providing stabilization with an accuracy of 0.01 °C. A fluidic system composed of a five-channel polydimethylsiloxane (PDMS) microfluidic cell (channel dimensions = 1.25 mm width x 500 µm height), a syringe pump (Darwin, Paris, France) and a 6-port injection valve (VICI; Texas, US), guarantee a continuous flow rate of the running buffer and the injections of the different solutions (**Figure S1C**).

2.2. BiMW sensor chips cleaning and silanization

The biosensor employs Si_3N_4 sensor chips, which must be cleaned and conditioned before custom biofunctionalization. First, the chips were cleaned by sonicating them sequentially in acetone, ethanol, and water for 5 min, followed by a 10 min incubation in 1:1 (v/v) methanol/hydrochloric acid (MeOH/HCl) solution to remove organic contamination. The chips were rinsed with milli-Q H_2O and dried under an N_2 stream. Then, a layer of active hydroxyl group was generated on the surface by O_2 plasma activation (Electronic Diener, Ebhausen, Germany) for 5 min at 45 sccm gas flow, followed by immersion in a 15 % HNO_3 solution at 95 °C for 25 min. The chips were immediately functionalized with APTES, following the protocol previously described by Huertas et al. [3]. In brief, the activated sensor chip was immersed into a toluene solution containing APTES 1 % (v/v) and DIPEA 0.3 % (v/v) for 1 h under an N_2 atmosphere. DIPEA was employed to catalyze the silanization reaction in the absence of water molecules. After the incubation, the sensor chips were rinsed with ethanol and dried with an N_2 stream. Then, a curing step was performed at 110 °C for 1 h in a conventional oven. Finally, APTES-modified sensor chips, displaying amino groups, were left to react with 20 mM PDITC solution (prepared in DFM containing 10 % anhydrous pyridine) for 1 h in darkness at room temperature. PDITC has two isothiocyanate (R-NCS) groups that can interact with primary amine groups through thiourea bonds (being a suitable homobifunctional cross-linker) and with thiol groups through the formation of thiocarbamate bonds.

3. Data analysis

The biosensor data were analyzed and processed using Origin 8.0 software (OriginLab, Massachusetts, US). Data and statistical analysis (i.e. Mann-Whitney, Kruskal-Wallis, ROC curves, and correlation tests) were performed using Graphpad Prism (Graphpad Software, Inc., California, US). The phase variation ($\Delta\varphi$) considered for the measurements was the one observed after signal stabilization, once all the samples had completely passed through the sensor chip (i.e. t ~3000 s for probe immobilization and t ~ 2500 s for detection assay in plasma). Calibration curves were obtained by evaluating different concentrations of the microRNA in triplicate and they were plotted as mean sensor signal ($\Delta\varphi$) and its

standard deviation (SD) versus microRNA concentration. Log-transformed variables were considered. Log (x)-log (y) plots were used for representation and the data were fitted to a linear equation (eq.1):

$$\log((\Delta\varphi) = A + b \cdot \log([miRNA]) \qquad \text{(eq. 1)}$$

The limit of detection (LOD) for microRNA-21-5p was calculated as the concentration corresponding to a blank signal plus three times its standard deviation. The coefficients of variation were obtained as the ratio of the standard deviation of the mean, expressed in percentages (% CV).

4. Figures

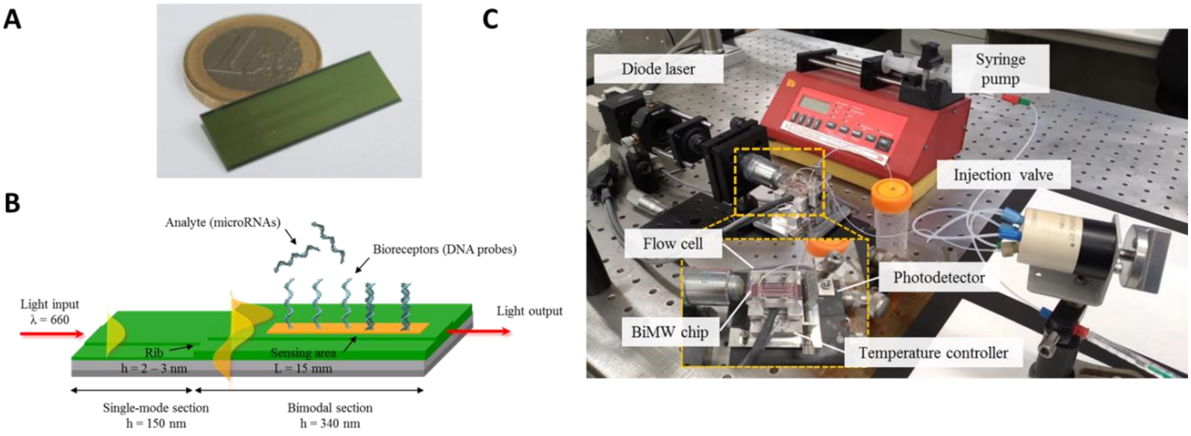

Figure S1. BiMW nanophotonic interferometer sensor. A) Photograph of a BiMW sensor chip. B) Schematic representation of the BiMW interferometer main characteristics and the working principle. C) Photograph of the BiMW set-up.

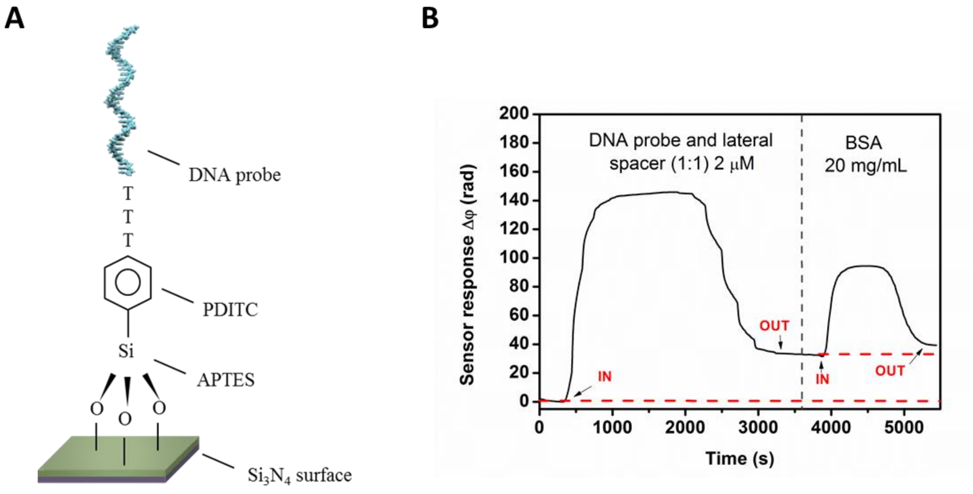

Figure S2. Si_3N_4 BiMW surface biofunctionalization. A) Scheme of the functionalization process performed for microRNA-21-5p detection. B) Real-time sensorgram of the DNA probe / lateral spacer (SH-PEG-COOH) immobilization and BSA blocking, over a PDITC-APTES activated surface.

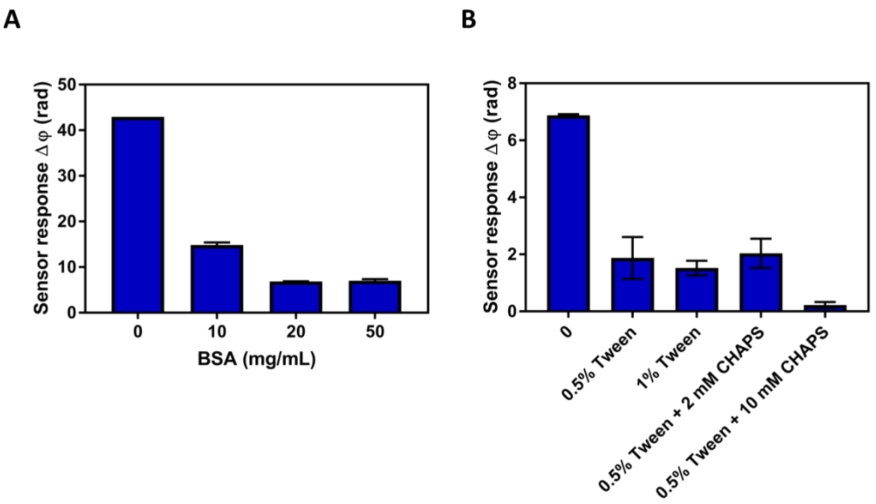

Figure S3. Effect of antifouling surfaces for plasma analysis. A) Sensor signals obtained from human plasma after covering the sensor surface with different concentrations of BSA blocking agent. B) Sensor signals obtained from human plasma after blocking the sensor surface with BSA 20 mg mL^{-1} and different combinations of detergents Tween 20 and CHAPS in the running buffer.

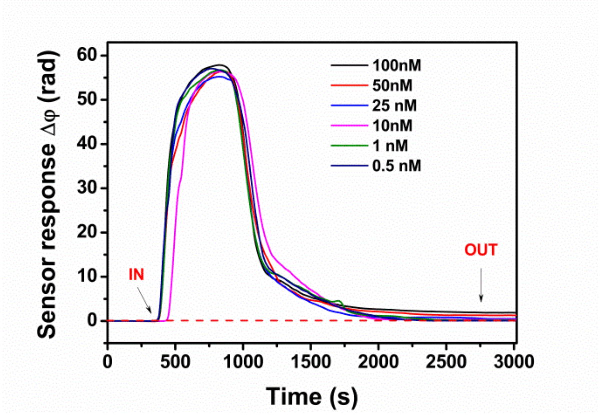

Figure S4. Real-time sensorgrams showing the specific interaction of microRNA-21-5p spiked in human plasma over DNA biofunctionalized chips. The bulk signal observed during sample injection results from the difference in the refractive index between the plasma and the running buffer (SSC-P (SSC 2.5X + 0.5 % Tween 20 + 10 mM CHAPS).

5. Lung cancer clinical samples

Table S1. Plasma samples (n=40) analyzed with qRT-PCR and the BiMW biosensor

Samples	Cancer stage[1]	T[*]	N[+]	M[ǂ]	Fold Change ($2^{-\Delta\Delta Ct}$)	BiMW (rad)
PLASMA 1	IIIA	3	2	0	1.12	4.74
PLASMA 2	IIIA	2	2	0	2.90	2.71
PLASMA 3	IIIA	3	2	0	1.40	3.77
PLASMA 4	IIIA	3	2	0	3.72	5.77
PLASMA 5	IIIB	4	2	0	3.14	4.27
PLASMA 6	IIIB	3	2	0	5.86	2.98
PLASMA 7	IIIB	3	2	0	3.12	3.35
PLASMA 8	IIIB	3	3	0	4.44	3.90
PLASMA 9	IIIA	2	2	0	4.85	4.46
PLASMA 10	IIIB	4	2	0	1.52	2.85
PLASMA 11	IIIB	4	2	0	6.52	3.33
PLASMA 12	IV	3	3	1	0.66	3.04
PLASMA 13	IV	4	3	1	7.54	2.34
PLASMA 14	IIIB	4	2	0	1.12	3.01
PLASMA 15	IIIB	4	3	0	4.49	4.58
PLASMA 16	IV	4	2	1	2.84	2.45
PLASMA 17	IIIB	4	2	1	3.68	2.66
PLASMA 18	IV	2	2	1	7.05	3.89
PLASMA 19	IIIB	4	2	0	5.03	12.88
PLASMA 20	IV	4	2	1	13.29	8.64
CONTROL 1					1.05	4.45
CONTROL 2					0.46	1.62
CONTROL 3					1.65	1.05
CONTROL 4					1.49	2.27
CONTROL 5					1.89	1.23
CONTROL 6					1.03	2.66
CONTROL 7					0.95	2.69
CONTROL 8					0.93	3.25
CONTROL 9					1.70	2.33
CONTROL 10					1.08	3.02
CONTROL 11					0.59	2.18
CONTROL 12					0.88	2.72
CONTROL 13					0.42	2.08
CONTROL 14					0.70	2.03
CONTROL 15					0.90	1.89
CONTROL 16					0.20	3.50
CONTROL 17					0.42	2.41
CONTROL 18					7.03	2.17
CONTROL 19					1.45	2.63
CONTROL 20					2.21	2.40

[1]Classification established according to the TNM system
[*]T refers to the size and extent of the primary tumor (Scale 1-4, 1-inner layer, 2-muscle layer, 3-into outer lining, 4-through outer lining)
[+]N refers to the number of nearby lymph nodes invasion (Scale 1-3, 1- none, 2-three, 3-four or more)
[ǂ]M refers to whether cancer has metastasized to other parts of the body (0= no metastasis; 1= metastasis)

6. References

[1] Zinoviev, K. E.; González-Guerrero, A. B.; Domínguez, C.; Lechuga, L. M. Integrated bimodal waveguide interferometric biosensor for label-free analysis. *J. Light. Technol.* **2011**, *29* (13), 1926-1930.

[2] Bassols-Cornudella, B.; Ramirez-Priego, P.; Soler, M.; Estévez, M.-C.; Luis-Ravelo, H. J. D.; Cardenosa-Rubio, M.; Lechuga, L.M. Novel Sensing Algorithm for Linear Read-out of Bimodal Waveguide Interferometric Biosensors. *J. Light. Technol.* **2022**, *40*(1), 237-244.

[3] Huertas, C.S.; Fariña, D.; Lechuga, L.M. Direct and Label-Free Quantification of Micro-RNA-181a at Attomolar Level in Complex Media Using a Nanophotonic Biosensor. ACS Sensors **2016**, 1 (6), 748–756. https://doi.org/10.1021/acssensors.6b00162.
